# Simultaneous Recognition of Dopamine and Uric Acid in the Presence of Ascorbic Acid via an Intercalated MXene/PPy Nanocomposite

**DOI:** 10.3390/s21093069

**Published:** 2021-04-28

**Authors:** Qiannan You, Zhongyang Guo, Rui Zhang, Zhimin Chang, Mingfeng Ge, Qian Mei, Wen-Fei Dong

**Affiliations:** 1 School of Biomedical Engineering (Suzhou), Division of Life Sciences and Medicine, University of Science and Technology of China, Hefei 230026, China; youqn@sibet.ac.cn (Q.Y.); iszy.guo@foxmail.com (Z.G.); zhangrui@sibet.ac.cn (R.Z.); 2 Suzhou Institute of Biomedical Engineering and Technology, Chinese Academy of Sciences, Suzhou 215163, China; changzm@sibet.ac.cn (Z.C.); gemf@sibet.ac.cn (M.G.)

**Keywords:** simultaneous recognition, dopamine, uric acid, MXene/PPy nanocomposite

## Abstract

Two-dimensional (2D) MXenes have shown a great potential for chemical sensing due to their electric properties. In this work, a Ti_3_C_2_T_x_/polypyrrole (MXene/PPy) nanocomposite has been synthesized and immobilized into a glassy carbon electrode to enable the simultaneous recognition of dopamine (DA) and uric acid (UA) under the interference of ascorbic acid (AA). The multilayer Ti_3_C_2_T_x_ MXene was prepared via the aqueous acid etching method and delaminated to a single layer nanosheet, benefiting the in-situ growth of PPy nanowires. The controllable preparation strategy and the compounding of PPy material remain great challenges for further practical application. A facile chemical oxidation method was proposed to regulate magnitude and density during the forming process of PPy nanowire, which promotes the conductivity and the electrochemical active site of this as-prepared nanomaterial. The MXene/PPy nanocomposite-modified electrode exhibited the selective determination of DA and UA in the presence of a high concentration of AA, as well as a wide linear range (DA: 12.5–125 μM, UA: 50–500 μM) and a low detection limit (DA: 0.37 μM, UA: 0.15 μM). More importantly, the simultaneous sensing for the co-existence of DA and UA was successfully achieved via the as-prepared sensor.

## 1. Introduction

Two-dimensional (2D) materials, which usually have an anisotropy of electrical characteristics, are promising candidates for use as electrode modified materials [1,2,3]. The 2D materials are restricted to two-dimensional planes due to their carrier migration and heat diffusion, making these materials exhibit many peculiar properties [4]. MXene, a new family of two-dimensional metal carbides, nitrides, and carbonitrides, has been a promising material in energy storage, membrane separation, electrochemical biosensors, etc. [5,6,7]. The formula of MXene is M_n+1_X_n_T_x_, wherein M represents the early transition metal, X is carbon and/or nitrogen, T_x_ represents surface functional groups (including oxygen (=O), hydroxyl (–OH), or fluorine (–F)), and n ranges from 1 to 3 [8,9]. Ti_3_C_2_T_x_ was generated by etching Al from an Ti_3_AlC_2_ MAX precursor [10,11], which exhibits unique electrical properties with a large surface area and is capable of using electrocatalyst analytes to construct chemical sensors [12,13,14]. Alongside this, the functional groups of Ti_3_C_2_T_x_ facilitate the subsequent chemical functionalization.

Polypyrrole (PPy) is a member of the conducting polymer family, which is arousing increasing attention owing to its high conductivity, good biocompatibility, and ease of synthesis [15,16]. Recently, conducting polymers and carbon-based nanomaterials have become promising candidates for use as electrode materials for electronic sensors. The performance of the conducting polymers being used directly as electrode materials can be optimized by a reasonable design of its synthesis and the regulation of its microstructure [17]. Compared with other conductive polymers, PPy possesses commercial application prospects in numerous applications, and can be used as a solid electrolytic capacitor, an anode material for secondary batteries, medical material, and an electrochemical sensor, among others [18,19,20,21]. The pyrrole monomer can be oxidized to polypyrrole via chemical oxidation or the electrochemical polymerization method [22,23]. However, the α and β positions of the pyrrole monomer are easy to cross-link, since they possess similar polymerization capabilities during the polymerization process (such as forming granular polypyrroles) and result in the poor conductivity of PPy [24,25]. Hence, the controllable synthesis procedure for certain and regular micromorphology is essential to the sensing performance of electrochemical materials.

Dopamine (DA), a catecholamine neurotransmitter secreted by the human brain, is an extensive participant in the nervous system, coordinating the function of the pituitary gland to secret hormones [26,27]. Uric acid (UA) is the metabolite of purine in vivo, which is closely related to kidney stones, diabetes, coronary heart disease and hypertension, etc. [28]. Performing accurate trace recognition of DA and UA is of great importance for scientific research and disease prevention. Currently, traditional detection techniques, including High-Performance Liquid Chromatography (HPLC), phosphotungstic acid reduction, and ultraviolet spectrophotometry, are limited to in-situ and real-time detection applications due to their expensive cost and long detection period [18,29,30]. When compared to other detection technologies, electrochemical sensors have advantages in terms of their accurate, fast and online DA and UA monitoring capabilities. However, the oxidation potentials of DA and UA are adjacent to that of ascorbic acid (AA), which leads to an overlap on the ordinary electrode from these three organic molecules during the electrochemical detection process [31]. So far, the modified electrode nanomaterials (including polymers [32,33], carbon-based nanomaterials [33,34], noble metals, and other metal oxides [35,36]) have been applied to improve catalytic selectivity for small molecules. The decoration of polymers with carbon-based nanomaterials is a promising method to develop a new strategy towards the simultaneous sensing of DA and UA.

**Figure 1:**
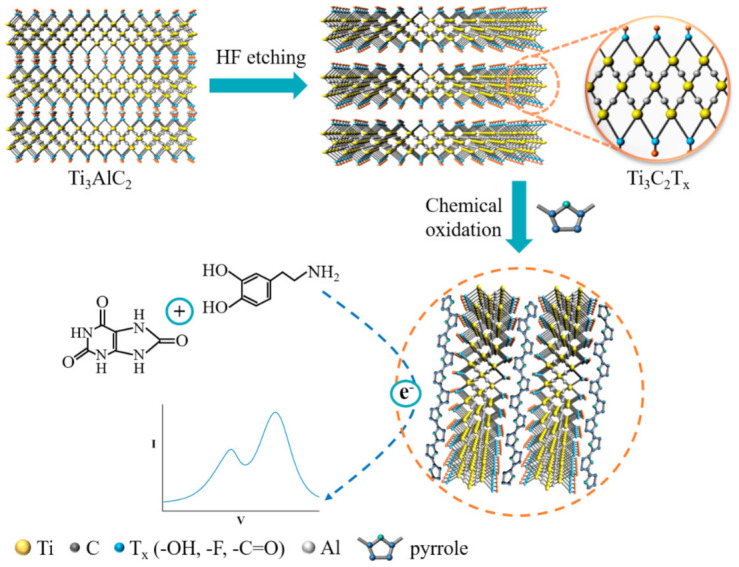
A schematic illustration of the synthesis of the MXene/PPy nanocomposite.

Herein, a novel chemical sensor based MXene/PPy nanocomposite was constructed via a simple chemical oxidation method. As shown in Figure 1, the Ti_3_C_2_T_x_ nanosheets were delaminated using hydrofluoric acid to etch away the aluminum atoms. PPy nanowires with diameters of ~200 nm were then composited into MXene single-layer nanosheets, which were regulated during the oxidation process. This as-prepared MXene/PPy nanocomposite material-modified electrode was applied towards the simultaneous sensing of DA and UA in the presence of AA. Due to the synergistic effects of conductive MXene and PPy with a well-defined nanowire structure, the nanocomposite modified electrode showed a wide detection linear range and a low detection limit.

## 2. Experimental

### 2.1. Materials

Pyrrole (C_4_H_4_NH), Methyl orange (MO) and Lithium fluoride (LiF) were purchased from Shanghai Aladdin Biochemical Technology Co., Ltd., Shanghai, China. Iron (III) chloride (FeCl_3_) was obtained from Sigma-Aldrich, Shanghai, China. Titanium aluminum carbide (Ti_3_AlC_2_) powders were purchased from 11 technology Co., Ltd., Changchun, Jilin province, China, and Hydrochloric acid (HCl) was obtained from Shanghai Lingfeng Chemical Reagent Co., Ltd., Shanghai, China. Dopamine (DA) and ascorbic acid (AA) were obtained from Shanghai Saan chemical technology Co., Ltd., Shanghai, China. Uric acid (UA) was purchased from Alfa Aesar Chemical Co., Ltd., Shanghai, China. All of the chemicals were directly applied without any further purification. The deionized water was applied for all solution preparations and washing steps.

### 2.2. Synthesis of Ti_3_C_2_T_x_ Powder

The synthesis of Ti_3_C_2_T_x_ was based on the aqueous acid etching method. 0.666 g LiF was added into 6 M HCl (10 mL) and stirred at room temperature for 40 min. Then, the Ti_3_AlC_2_ powders (1 g) were dispersed into the mixture solution to react at 35 °C for 24 h. The resulting suspension was washed alternately with ethanol and deionized water until the pH reached 6, and was then dried overnight in a vacuum drying oven for a subsequent alternate preparation method.

### 2.3. Synthesis of MXene/PPy Nanomaterial

MXene/PPy was prepared through a chemical oxidation process. Ti_3_C_2_T_x_ powders were dispersed in deionized water and treated with powerful ultrasonication to delaminate them for use as nanosheets. Then, the Ti_3_C_2_T_x_ nanosheets were mixed into a solution of 0.1 M FeCl_3_ and 0.01 M MO and stirred for 30 min at room temperature. At last, 60 μL pyrrole was added into the sample, followed by stirring at room temperature for 24 h. The final MXene/PPy nanocomposite was created via centrifugation and vacuum drying in succession.

### 2.4. Preparation of the MXene/PPy Modified Electrode

The synthesized MXene/PPy nanocomposite was dispersed in deionized water and stirred homogenously. Then, 10 μL of the synthesized solution was dropped on the surface of the glassy carbon electrode (GCE, Φ = 3 mm) and the modified electrode was used as the working electrode.

### 2.5. Characterizations and Electrochemical Measurements

The morphology of the samples was characterized using a field emission scanning electron microscope (FESEM) (S4800, Hitachi, Japan). The sample structure and element analyses were performed using X-ray diffraction (XRD) (Cu–Ka line (0.15419 nm), D/MAX 2500 V/PC), Fourier transforms infrared (FT-IR) (Thermo Electron Corporation, Nicolet-8700, USA), X-ray photoelectron spectroscopy (XPS, ESCLAB MKII), and transmission electron microscopy (TEM). UV-vis spectra were obtained using a UV-vis adsorption spectrophotometer (Agilent Cary 300 Scan). All of the electrochemical measurements were carried out using an electrochemical workstation (CHI 660E, Shanghai Chenhua Instrument Co., Ltd., Shanghai, China).

All electrochemical measurements were carried out in a three-electrode system. The as-prepared MXene/PPy nanocomposite was used as the working electrode. A Pt wire served as the counter electrode, and an Ag/AgCl (saturated KCl) electrode served as the reference electrode. The electrochemical properties of the working electrode were characterized by cyclic voltammetry (CV), electrochemical impedance spectroscopy (EIS) and differential pulse voltammetry (DPV).

## 3. Results and Discussion

### 3.1. Characterization of MXene/PPy

**Figure 2:**
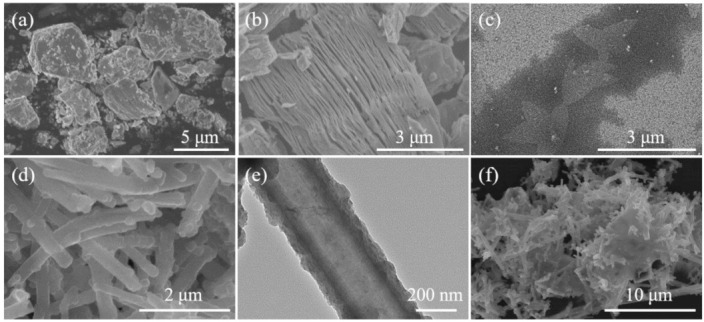
The synthesis of the MXene/PPy nanocomposite. A FESEM image of (**a**) Ti_3_AlC_2_ powders, (**b**) Ti_3_C_2_T_x_ powders, (**c**) Ti_3_C_2_T_x_ nanosheets, and (**d**) PPy nanowires; (**e**) a TEM image of the PPy nanowire; (**f**) a FESEM image of the MXene/PPy nanocomposite.

As illustrated by our design in Figure 1, the regular morphology of the PPy nanowire was controlled by the size of the Ti_3_C_2_T_x_ MXene nanosheet during the oxidation process. The Ti_3_C_2_T_x_ MXene was first etched into a layered accordion-like structure, and the morphology of the precursor Ti_3_AlC_2_ MAX powders and the delaminated Ti_3_C_2_T_x_ MXene were characterized in Figure 2a–c. The Ti_3_C_2_T_x_ exhibited a well-stacked multilayer structure after etching of the Al atom layers from the precursor Ti_3_AlC_2_ with HF treatment, as seen in Figure 2b. Then, the MXene nanosheets were easily produced by sonication. A certain amount of Ti_3_C_2_T_x_ powders was dispersed in deionized water followed by sonication to prepare a thin layer of MXene nanosheets. As shown in Figure 2c, the single-layer MXene nanosheets with a dimension of ~1 μm can be obtained after sonication and delamination for 45 min. As for our synthesis in Figure 2d, large scale and homogeneous PPy nanowires were successfully prepared via the chemical oxidation method. The growth mechanism of the PPy nanowire was demonstrated in Figures S1 and S2, revealing the decisive function of the oxidant and demonstrating a template for the regulation of the nanostructure of PPy (Supplementary Materials). The nanostructure of the PPy nanowire was further examined using TEM, which possessed a uniform diameter of ~200 nm under the optimal fabrication conditions (Figure 2e). With the addition of the Ti_3_C_2_T_x_ nanosheets, uniform nanowires were interspersed in stacked nanosheet layers (Figure 2f), showing the successful insertion of the PPy nanowires.

**Figure 3:**
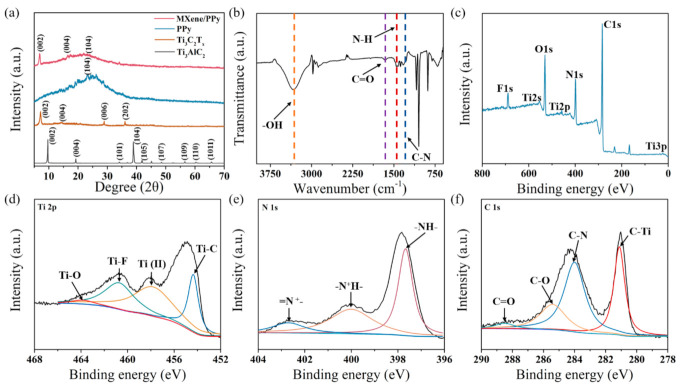
(**a**) The XRD patterns of Ti_3_AlC_2_-MAX, Ti_3_C_2_T_x_-MXene, PPy and MXene/PPy powders; (**b**) the FT-IR diagram of MXene/PPy powder; (**c**–**f**) the XPS spectra of MXene/PPy, Ti 2p, N 1s, and C 1s in the MXene/PPy nanocomposite, respectively.

Figure 3a shows the XRD patterns of Ti_3_AlC_2_-MAX, Ti_3_C_2_T_x_-MXene, PPy, and MXene/PPy, respectively. When compared with their MAX precursors, the characteristic peaks located at 7.1°, 14.4°, 28.9°, and 36.1° are assigned to the (002), (004), (006), and (202) facets of Ti_3_C_2_T_x_, respectively. This confirms that the Ti_3_C_2_T_x_ nanosheet was successfully converted after etching Al from the Ti_3_AlC_2_ powder. Alongside this, the typical PPy peak was observed at about 24°, which indicates an average chain of ~4.38 Å for PPy. When the PPy nanowire structures were formed on the Ti_3_C_2_T_x_ nanosheets, the diffraction pattern in Figure 3a indicated the well-defined lattice structure of this nanocomposite. Furthermore, FT-IR and XPS spectroscopy were applied to characterize the functional group of the MXene/PPy nanocomposite. The absorption peaks of Ti_3_C_2_T_x_ can be observed at 3357 cm^−1^ and 1645 cm^−1^, which correspond to -OH and -C=O, respectively (Figure 3b). The chemical composition of this nanocomposite before and after PPy oxidation was verified by the obtained XPS spectra. According to the characterization results in Figure 3c and Figure S3b, the PPy peak appeared under the existence of Ti_3_C_2_T_x_, and the other peaks were rarely changed. In the high resolution of the XPS spectrum for the Ti element in the MXene/PPy nanocomposite (Figure 3d), four peaks appeared at 454.3 eV, 457.9 eV, 460.7 eV, and 463.5 eV, which correspond to Ti-O, Ti (II), Ti–F, and Ti–O, respectively. The detailed combination of this nanocomposite was analyzed using the XPS results for N 1s and C 1s. In Figure 3e, the binding energies of 397.6 eV, 400.1 eV, and 402.7 eV represented -NH-, -N^+^H-, and =N^+^-, respectively. Additionally, the C-N located at 284.1 eV from the C 1s spectrum indicated the interactions between the pyrrole monomers and the MXene nanosheets (Figure 3f). The above results demonstrated that the delaminated Ti_3_C_2_T_x_ nanosheets could maintain their crystalline structure during the oxidation of PPy.

### 3.2. Electrochemical Behaviors of MXene/PPy Modified Electrode

**Figure 4:**
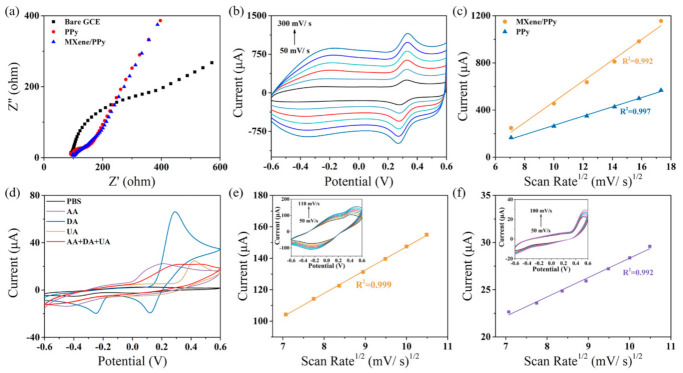
(**a**) Nyquist plots of bare, PPy modified, and MXene/PPy modified electrodes in the presence of 10 mM (Fe(CN)_6_)^3-/4-^ containing 0.1 M KCl; (**b**) the CV curves of the MXene/PPy modified electrode with different scan rates (50, 100, 150, 200, 250, and 300 mV/s) in 10 mM K_3_(Fe(CN)_6_) containing 3 M KCl; (**c**) the calibration curves of current peak vs. the square root of the scan rate for PPy modified and MXene/PPy modified electrodes; (**d**) the CV responses of the MXene/PPy modified electrode for 1 mM AA, 50 μM DA, and 100 μM UA in 0.05 M PBS; (**e**,**f**) the calibration curves of the current peak vs. the square root of the scan rate for the MXene/PPy modified electrode in 0.05 M PBS containing 50 μM DA and 100 μM UA, respectively (the insert shows the CV curves of the MXene/PPy modified electrode with different scan rates (50, 60, 70, 80, 90, 100, and 110 mV/s)).

The electrochemical behaviors of the MXene/PPy nanocomposite were investigated in Figure S4a. A cyclic voltammetry (CV) test was applied to characterize the electrochemical conductivity of the bare glassy carbon electrode (GCE), the pure PPy modified electrodes, and the MXene/PPy modified electrodes in 0.05 M PBS (Figure S4a). Compared with the pure PPy modified electrode, the MXene/PPy modified electrode showed two pairs of redox peaks (oxidation peaks at 0.21 V and −0.48 V, reduction peaks at −0.17 V and 0.31 V), which correspond to the electrocatalytic processes of MXene and PPy, respectively. It is obvious that the peak current exhibited a remarkable increase during both oxidation and reduction, indicated by the significant enhancement of the electron transfer rate via the introduction of the MXene nanosheet. EIS was used to evaluate the electron transfer resistance (R_ct_) of the as-prepared electrodes in Figure 4a. The Rct values of the bare GCE, the pure PPy modified electrodes, and the MXene/PPy modified GCE electrodes were calculated to be 109.63 Ω, 23.09 Ω, and 15.34 Ω, respectively. The above results demonstrate that the participation of MXene enables the improvement conductivity of the PPy. The effective surface area of these modified electrodes was further investigated through CV with different scan rates ranging from 50 to 300 mV/s (Figure 4b). As shown in Figure 4c, the linear curves that present the relationship between the oxidation peak current, the square root of the scan rate, and the effective surface area can be calculated in accordance with the Randles–Sevcik equation [37,38,39]:

$$\frac{I_{p}}{\nu^{1/2}}=\left(2.69\times 10^{5}\right)n^{3/2}D_{0}^{1/2}C_{0}^{*}A$$

In this formula, A is the specific surface area and I_p_ and v^1/2^ represent the peak current and square root of the scan rate, respectively. $C_{0}^{*}$ is the concentration of the detection molecule, n is the number of electrons transferred in this redox process, and D_0_ is the molecular diffusion coefficient. A proportional relationship between I_p_/v^1/2^ and A is the calculated basis of the effective surface area, in addition to the constant values (n, D_0_ and C_0_). The peak currents and the square roots of the scan rates exhibited a linear relationship, and the I_p_/v^1/2^ is a fixed value that represents the Randles’ slope. According to the calibration curves in Figure 4c and Figure S4b, the Randles’ slopes of MXene/PPy and the pure PPy modified electrodes were calculated to be 88.11 ± 4.05 and 39.39 ± 1.12, respectively (Table 1). Therefore, it could be inferred that the effective surface area of the MXene/PPy (2.87 ± 0.13 cm^2^) was increased to about 2.24 times that of the pure PPy (1.28 ± 0.04 cm^2^) and is thus able to provide more electrocatalytic sites for sensing (Table S1).

CV measurement was further utilized to determine the optimal working potential of the MXene/PPy modified electrode for DA and UA sensing. The response results of the as-prepared electrode toward DA, UA, and AA were shown in Figure 4d. It is clear that the electrocatalytic reaction of these analytes can effectively separate the overlapped oxidation peak. For DA, an oxidation peak appeared at 0.29 mV, indicating the electrocatalytic effect of DA on o-dopaminoquinone at a MXene/PPy nanocomposite modified electrode. In the case of UA, the oxidation current peak increased at 0.42 mV with the injection of 100 μM UA, implying that the reversible reaction of UA to diamine is intermediate. However, the injection of AA also altered the redox behavior of the as-prepared electrode, which indicates that the modification was responsive to the individual AA molecule. According to the above characterization results, the CV method was applied to allow for the separate recognition of DA, UA, and AA. The result in Figure 4d indicates that the MXene/PPy modified electrode can recognize DA and UA in the presence of AA and that the oxidation potential of both DA and UA show a slight offset towards positive potential. In addition, there is no obvious oxidant peak in AA with the simultaneous addition of DA, UA, and AA. Therefore, the as-prepared MXene/PPy modified nanocomposite can only respond to DA and UA in the presence of AA. Then, the sensing mechanisms of DA and UA on the as-prepared electrode surface were investigated using CV scans from 50 mV/s to 110 mV/s in 0.05 M PBS (Figure 4e,f). The cyclic voltammetry results were fitted as shown in the insert; the oxidation peak currents of both DA and UA demonstrated a linear relationship with the square root of the scan rate. The simulation results present the finding that the catalytic oxidation processes of DA and UA are controlled via the diffusion process, and the as-prepared sensor is able to quantitatively determine DA and UA.

### 3.3. Sensing Performance of MXene/PPy Modified Electrode

**Figure 5:**
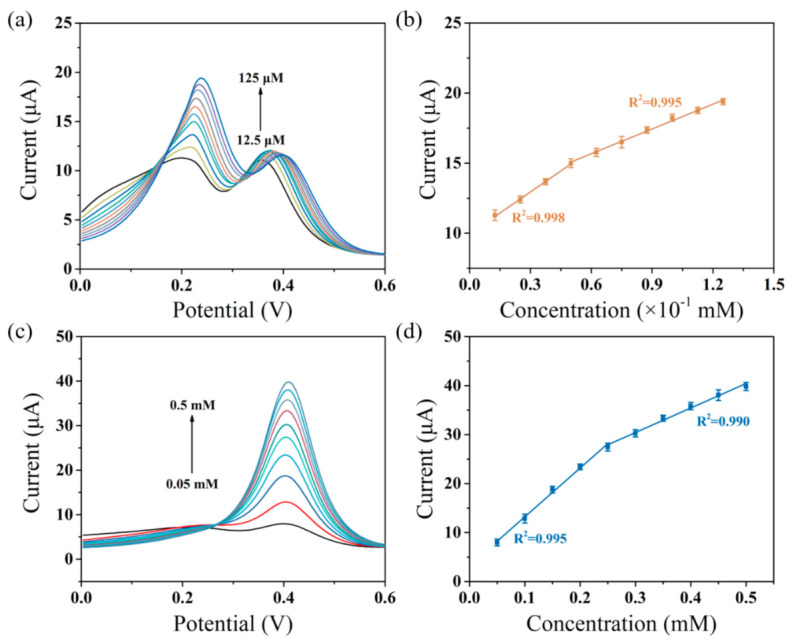
(**a**) Differential pulse voltammetry (DPV) responses of the MXene/PPy modified electrode with different DA concentrations in the presence of 1 mM AA and 100 μM UA; (**b**) a linear calibration curve for the current response of DA; (**c**) DPV responses of the MXene/PPy modified electrode with different UA concentrations in the presence of 1 mM AA and 50 μM DA; (**d**) a linear calibration curve for the current response of UA.

As designed, the composite of Ti_3_C_2_T_x_ and polypyrrole provides a substantial basis for the simultaneous determination of DA and UA in the presence of AA. To ensure the optimal potential of the DA and UA sensing process, the DPV method was applied to characterize the response current of the MXene/PPy-based sensor with the successive injection of variation concentration substances. Figure 5 shows two independent potentials at 0.23 mV and 0.40 mV, which correspond to the oxidation of DA and UA, respectively. As observed in Figure 5a, the peak current was increased after each addition of DA until the concentration of DA reached 125 μM in 0.05 M PBS containing 1 mM AA and 100 μM UA. Apparently, the initial injection of UA aroused an obvious peak, while AA hardly responded. In addition, the same phenomenon (Figure 5c) can be observed with the concentration change of UA in the presence of 1 mM AA and 50 μM DA. As discussed above, the as-prepared sensor can independently sense DA and UA given the coexistence of AA, and resist mutual interference. According to the data in Figure 5a,c, the calibration lines of the peak current with the corresponding reactant concentration were simulated in Figure 5b,d, respectively. As shown in Figure 5b, the fitting linear equations of DA are I_DA_ = 9.928C_DA_ + 9.99 (R^2^ = 0.998, 12.5–50 μM) and I_DA_ = 5.977C_DA_ + 12.061 (R^2^ = 0.995, 50–125 μM), respectively (I and C represent the current intensity (μA) and substrate concentration (mM), respectively). Similarly, the linear calibration equation of UA can be fitted as I_UA_ = 99.008C_UA_ + 3.242 (R^2^ = 0.995, 50–250 μM) and I_UA_ = 50.16C_UA_ + 15.313 (R^2^ = 0.990, 250–500 μM), respectively. These results demonstrate that the as-prepared sensor can separately examine DA and UA with a wide linear range and high sensitivity.

**Figure 6:**
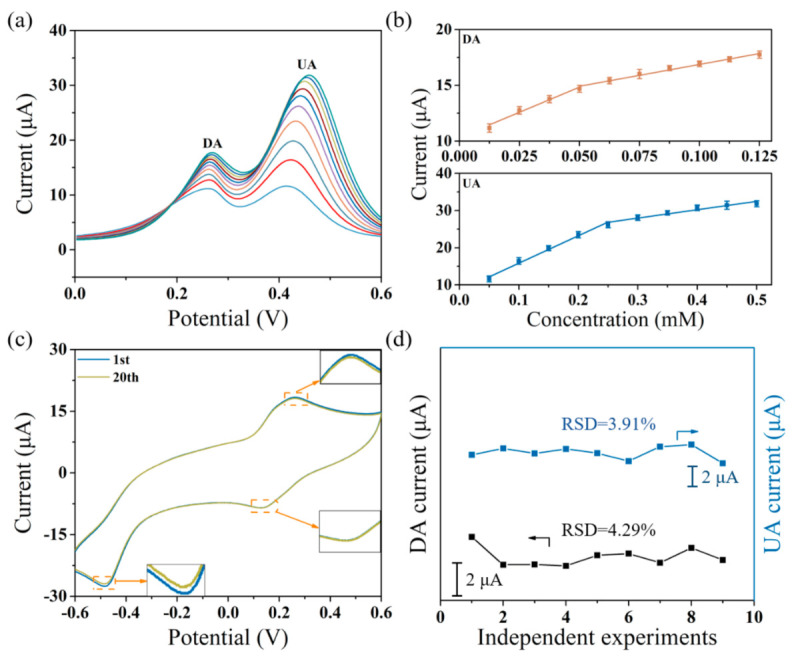
(**a**) DPV responses of the MXene/PPy modified electrode for the mixture containing DA (12.5–125 μM), UA (50–500 μM), and 1 mM AA; (**b**) the linear calibration curves for the current response of DA and UA; (**c**) the stability of the as-prepared sensor after scanning in 0.05 M PBS for 20 cycles; (**d**) the reproducibility of the as-prepared sensor in 0.05 M PBS containing 50 μM DA and 100 μM UA.

The synchronous sensing of DA and UA in the presence of AA was examined via the simultaneous injection of the two substances containing 1 mM AA on the MXene/PPy nanocomposite modified electrode. As shown in Figure 6a, the oxidation peaks of DA and UA were relatively independent, and the addition of AA had a hard time interfering with the current signal. The linear calibration curves of DA and UA were also fitted between the current signal and the substance concentration in Figure 6b (DA: 12.5–125 μM; UA: 50–500 μM). In addition, the detection limits of DA and UA can reach 0.37 μM and 0.15 μM, respectively (at an S/N of 3). Obviously, there were rare sensing performance differences between the separate and simultaneous detection of the two molecules. For evaluating the performance of the as-prepared sensor, electrochemical stability is an essential index, obtained through CV measurement. Examining a comparison of the first- and twentieth-times scan rates in Figure 6c, the tiny changes in the magnitude of the redox peaks indicate the excellent electrochemical stability of the MXene/PPy modified electrode for reactant sensing. Subsequently, the reproducibility of the as-constructed sensor was performed using nine MXene/PPy modified electrodes under the same conditions. The response signals were measured separately for the same addition of 50 μM DA and 100 μM UA. The relative standard deviations (RSD) of DA and UA in Figure 6d were calculated as 3.91% and 4.29%, respectively, revealing the excellent reproducibility of the sensor-based MXene/PPy nanocomposite.

The sensing performance of the MXene/PPy modified electrode was compared with those of other reported sensors in Table 1. According to the data from Table 1, the sensor-based MXene/PPy material exhibited a wider linear range and a lower detection limit than did most other reported sensors. Furthermore, the low detection limit of the as-prepared sensor also promises a great potential for practical applications.

## 4. Conclusions

In this work, we synthesized delaminated Ti_3_C_2_T_x_ through the aqueous acid etching method and in-situ complexed PPy nanowire successfully via chemical oxidation. The regular structure of PPy nanowires was effectively controlled via the intercalation in the nanosheets of Ti_3_C_2_T_x_-MXene. Under the optimized synthesis conditions, the composite material provides more active electrochemical sites owing to its larger effective surface area compared to pure PPy nanowire material. Meanwhile, the nanowire structure of PPy and the two-dimensional structure of Ti_3_C_2_T_x_ are conducive to the loading of target substances and the transfer of electrons. With a combination of these two promising materials, the as-prepared sensor exhibited a wide linear range and a low detection limit for the simultaneous sensing of DA and UA in the presence of AA and showed excellent stability and reproducibility. This work provides a new avenue for DA and UA recognition using two-dimensional materials and conductive polymer composite materials, and the designed electrochemical sensor has great potential in actual system monitoring.

## Figures and Tables

**Table 1:** Performance comparisons of various modified electrodes for the simultaneous determination of DA and UA.

Electrode Materials	Linear Range (μM)		Detection Limit (μM)		Reference
	DA	UA	DA	UA	
PGE	0.15–15	0.3–150	0.033	0.12	[27]
Graphene flowers/CFE	0.7–45.21	3.78–183.87	0.5	2	[40]
Hema/GCE	5–20	2.5–20	0.5	0.63	[41]
CuZEA/RGO/ GCE	0.1–19	20–200	0.041	11	[42]
Mesoporous carbon nanofiber-modified pyrolytic graphite electrode	0.05–30	0.5–120	0.02	0.2	[43]
Poly (DBF)	0.2–200	1.0–250	0.2	0.03	[44]
Reduced graphene oxide/GCE	0.5–60	0.5–60	0.5	0.5	[29]
MXene/PPy	12.5–125	50–500	0.37	0.15	Current Work

## Data Availability

Not applicable.

## Supplementary Materials

The following are available online at https://www.mdpi.com/article/10.3390/s21093069/s1. Figure S1: FESEM images of PPy with different concentration of oxidant, Figure S2: FESEM images of PPy with different concentration of MO, Figure S3: XPS spectra and UV-vis spectra, Figure S4: CV behaviors of bare, PPy and MXene/PPy modified electrode, Figure S5: Anti-interference and stability of MXene/PPy modified electrode, Table S1: Comparison of the electrochemical effective surface area of PPy modified and MXene/PPy modified electrode.
