# Supplementary material for: Simultaneous Recognition of Dopamine and Uric Acid in the Presence of Ascorbic Acid via an Intercalated MXene/PPy Nanocomposite
Source: Sensors (Basel). 2021 Apr 28;21(9):3069. doi: 10.3390/s21093069 (PMC8124802; doi:10.3390/s21093069)
Supplement: Supplementary file 1 [file sensors-21-03069-s001.zip › sensors-1114396-supplementary.pdf]

Supplementary Material

Simultaneous recognition of dopamine and uric acid in the presence of ascorbic acid via an intercalated MXene/PPy nanocomposite

Qiannan You[a,b], Zhongyang Guo[a,b], Rui Zhang[a,b], Zhimin Chang[b], Mingfeng Ge[b], Qian Mei[b,*], Wen-Fei Dong[b,*]

[a] School of Biomedical Engineering (Suzhou), Division of Life Sciences and Medicine, University of Science and Technology of China, Hefei 230026, P. R. China

[b] Suzhou Institute of Biomedical Engineering and Technology, Chinese Academy of Science, Suzhou 215163, P. R. China

Corresponding Author

*E-mail: qmei@sibet.ac.cn; wenfeidong@sibet.ac.cn

Tel: +86-512-6958-8307; Fax: +86-512-6958-8088.

Contents

Fig. S1 FESEM images of PPy with different concentration of oxidant 3

Fig. S2 FESEM images of PPy with different concentration of MO 4

Fig. S3 XPS spectra and UV-vis spectra 5

Fig. S4 CV behaviors of bare, PPy and MXene/PPy modified electrode 6

Fig. S5 Anti-interference and stability of MXene/PPy modified electrode 7

Tab. S1 Comparison of the electrochemical effective surface area of PPy modified and MXene/PPy modified electrode 8

Fig. S1 FESEM images of PPy with different concentration of oxidant

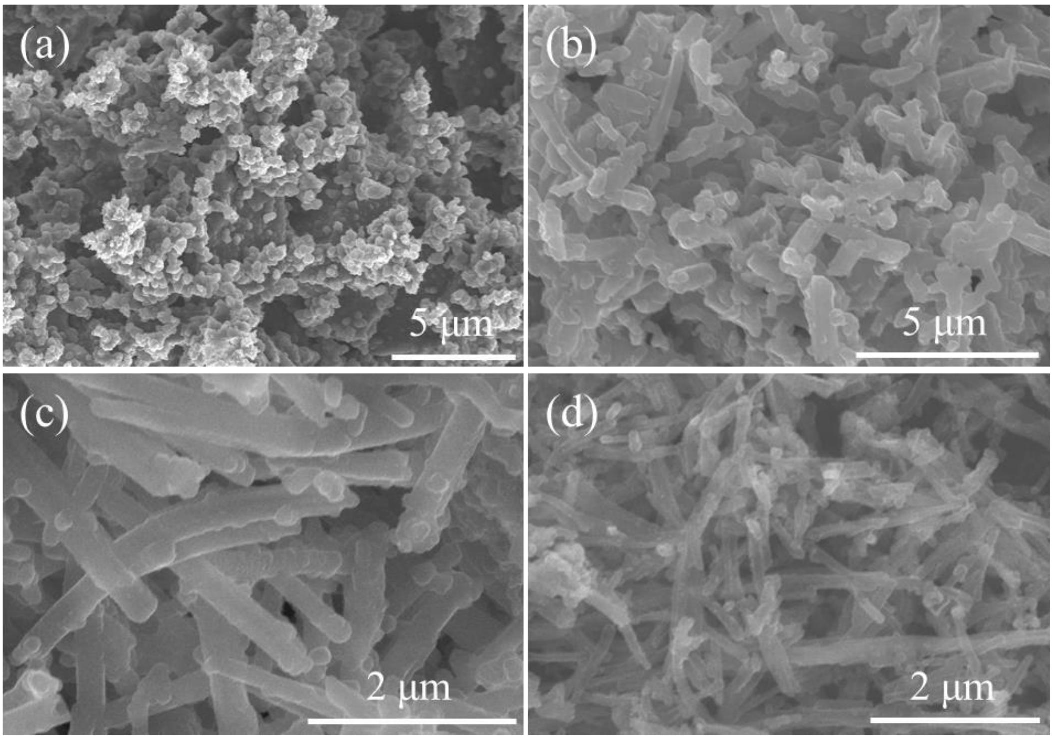

Fig. S1. FESEM images of PPy synthesized at 35 ºC for 24 h in a system containing 0.01 M MO and different concentrations of $FeCl_3$ (a) 0.01 M, (b) 0.03 M, (c) 0.1 M and (d) 0.15 M.

Fig. S2 FESEM images of PPy with different concentration of MO

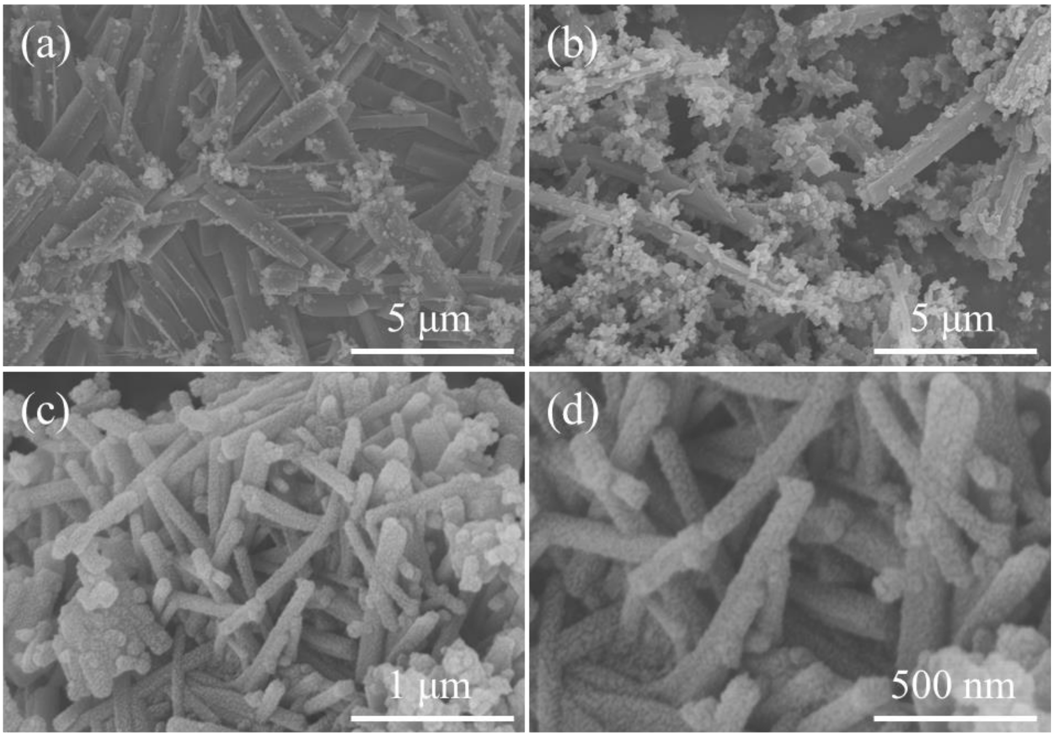

Fig. S2. FESEM images of PPy synthesized at 35 ºC for 24 h in the presence of different concentrations of MO (a, b) 0.005 M and (c, d) 0.01 M with 0.1 M $FeCl_3$.

Fig. S3 XPS spectra and UV-vis spectra

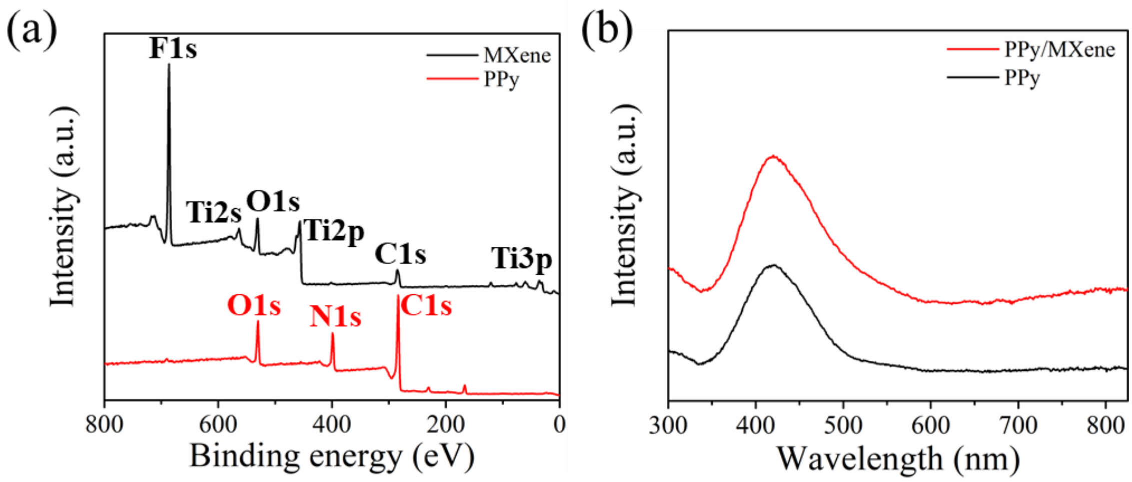

Fig. S3. (a) XPS spectra of PPy nanowire and MXene powder, respectively; (b) UV-vis spectra of PPy nanowire and MXene/PPy nanocomposite, respectively.

UV-vis adsorption spectra were applied for characterizing the spectroscopic property of pure PPy and MXene/PPy nanocomposite. The adsorption peak of PPy was observed at ~420nm, which should be ascribed to the π-π^* transition band. The spectrum shows a typical behavior of PPy, and the composite after introducing MXene slightly affected the structure of PPy.

Fig. S4 CV behaviors of bare, PPy and MXene/PPy modified electrode

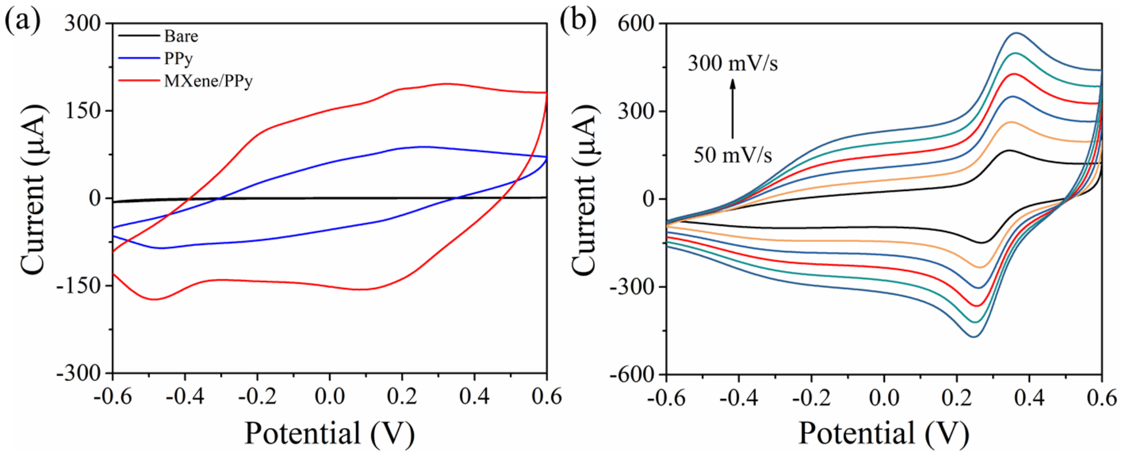

Fig. S4. (a) CV behaviors of bare, PPy modified and MXene/PPy modified GCE electrodes in PBS at a scan rate of 50 mV/s; (b) CV curves of PPy nanowire modified electrode in 0.05 M PBS at different scan rates from 50 to 300 mV/s.

Fig. S5 Anti-interference and stability of MXene/PPy modified electrode

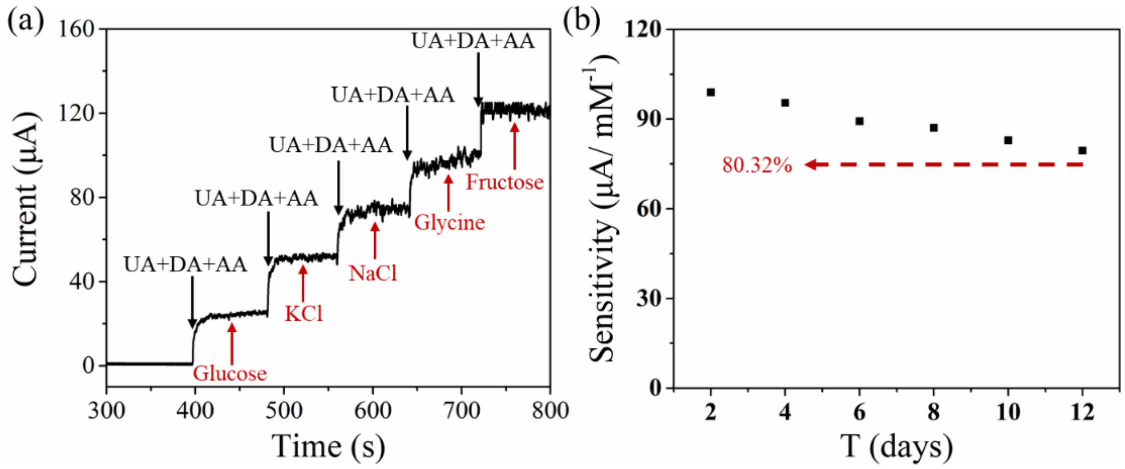

Fig. S5. Calibrated response with addition of 1 mM different substances (glucose, potassium chloride, sodium chloride, glycine and fructose) on MXene/PPy modified GCE electrode in 0.05 M PBS; (b) Usage stability test of the as-prepared MXene/PPy electrode for 12 days.

The long-term stability can be obtained by storing one electrode at 4 °C with the continuous tests for every two days. After 12 days usage, this sensor can still remain 80.32% of initial sensitivity, indicating the good usage stability.

Tab. S1 Comparison of the electrochemical effective surface area of PPy modified and MXene/PPy modified electrode

Tab. S1. The effective surface area of PPy modified and MXene/PPy modified electrode.

Electrode	Randle's slope ($\mu A/ (mV^{1/2} \cdot s^{1/2})$)	Effective surface area (cm^2)
PPy	39.39±1.12	1.28±0.04
MXene/PPy	88.11±4.05	2.87±0.13
